# Double-Layer Cubature Kalman Filter for Nonlinear Estimation †

**DOI:** 10.3390/s19050986

**Published:** 2019-02-26

**Authors:** Feng Yang, Yujuan Luo, Litao Zheng

**Affiliations:** 1 School of Automation, Northwestern Polytecnical University, Xi’an 710129, China; lyjnwpu@mail.nwpu.edu.cn (Y.L.); zhenglitao@mail.nwpu.edu.cn (L.Z.); 2 Key Laboratory of Information Fusion Technology, Ministry of Education, Xi’an 710129, China; 3 CETC Key Laboratory of Data Link Technology, No. 20 Institute of CETC, Xi’an 710000, China

**Keywords:** nonlinear estimation, deterministic sampling strategy, cubature Kalman filter, cubature particle filter

## Abstract

The cubature Kalman filter (CKF) has poor performance in strongly nonlinear systems while the cubature particle filter has high computational complexity induced by stochastic sampling. To address these problems, a novel CKF named double-Layer cubature Kalman filter (DLCKF) is proposed. In the proposed DLCKF, the prior distribution is represented by a set of weighted deterministic sampling points, and each deterministic sampling point is updated by the inner CKF. Finally, the update mechanism of the outer CKF is used to obtain the state estimations. Simulation results show that the proposed algorithm has not only high estimation accuracy but also low computational complexity, compared with the state-of-the-art filtering algorithms.

## 1. Introduction

Nonlinear estimation has been widely used in many fields, such as information fusion, computer vision, engineering, and economics [1,2,3,4]. Some classic estimation algorithms are put forward to deal with nonlinear systems. These filters can be classified as two categories: point estimation (e.g., the extended Kalman filter [5], unscented Kalman filter [6,7] and cubature Kalman filter [8,9,10]), and density estimation (e.g., the particle filter [11,12]). The former uses moment and density function to characterize variables [13] as the latter approximates posterior density function on the basis of bayesian formula [14]. The extended Kalman filter (EKF) [5] has been widely used in dealing with nonlinear systems. By using Taylor series expansion, it linearizes nonlinear system equations and can be applied in nonlinear systems. However, as it must obey the Gaussian distribution, in strongly nonlinear [15] and non-Gaussian systems, the EKF will perform poorly [16]. Based on unscented transform (UT), the unscented Kalman filter (UKF) [6,7] can obtain a higher filtering accuracy than the EKF. However, the weights of the sigma points in the UKF are prone to be negative in high-dimension systems, leading to numerical instability as well as low estimation accuracy. The cubature Kalman filter (CKF) [8,9,10] uses the spherical-radial cubature rule to generate some weighted sampling points to approximate integral in Bayesian estimation. It has estimation accuracy up to three order and is widely used in high order nonlinear systems [17]. Therefore, the CKF algorithm is mainly discussed in this paper. However, in non-Gaussian systems, the CKF will degrade. Further, it has numerical instability in the process of practical applications [18].

As the estimation accuracy of the conventional CKF only reaches three order, reference [19] proposed a high-order cubature Kalman filter in order to augment the estimation accuracy. In this filtering method, multiple integral and moment matching are respectively adopted to derive arbitrary-order spherical rule and the radial rule in the frame of points-based Gauss approximation. Consequently, a high-order spherical-radial cubature rule is attained and then employed to calculate Gaussian weighted integrals. As a result, the high-order cubature Kalman filter is established which can improve the estimation accuracy greatly. However, high estimation accuracy is accompanied by large computational load, especially in the problem with a high dimensionality [20]. It is unsuitable for systems which require high real-time performance accordingly [21]. The above methods still suffer from high measurement equation nonlinearity.

The square-root cubature Kalman filter (SRCKF) [18] adopts orthogonal triangular decomposition to avoid the root operation in matrix. As a result, it has merits such as better numerical instability than the CKF as well as high efficiency. The iterated cubature Kalman filter (ICKF) [22] combines the Gauss-Newton iterated method with the CKF, reducing the estimation error. The recursive update extended Kalman filter (RUEKF) [23] incorporates nonlinear measurements into a Kalman filter by applying the update gradually. Specifically, it recalculates Jacobian matrix and keeps the measurement nonlinear while updating [23]. One of the advantages is that its computational load is not excessive since the filter is free from the curse of dimensionality. Above all, it is not necessary to linearize the update equation. On the basis of the similar approach, reference [24] reported the recursive update cubature Kalman filter (RUCKF), in which statistical linearization technique based on sigma transformation is used [24]. The RUCKF does not rely on Jacobian matrix as its Kalman gain is computed by state prior distribution rather than state estimate.

In nonlinear system models with non-Gaussian noise, the particle filter (PF) [11,12], which is based on Bayesian theory and Monte Carlo method, is an effective method to deal with the problem of nonlinear estimation. In the PF algorithm, the probability distribution is approximated by a large number of random samples [25] for which, the weights and the positions of the samples are adjusted to approximate the posterior probability distribution function (PDF) on the basis of the measurement [26,27]. Since the method is not interfered by system linearity and not subject to the Gaussian distribution, it can deal with nonlinear, non-Gaussian system models [28]. However, as the key component of the PF is to approximate importance PDF by selecting particles, the performance of this method is affected by the curse of importance PDF. To solve this problem, a cubature particle filter (CPF) [29,30] has been proposed, where the estimation of the CKF is adopted as the proposal distribution. It greatly improves the performance as compared with the conventional PF. Nevertheless, the CPF suffers from heavy computational load as the result of the use of a large number of particles.

Cascade structure algorithm is used to increase the system efficiency in [31,32]. Reference [31] applied orthogonal genetic-algorithm (OGA) to a cascade finite impulse response (FIR) realization to obtain the multiplierless design. Reference [32] proposed a Map/INS/Wi-Fi integrated system based on a cascaded Particle/Kalman filter framework structure to decrease the system computational burden.

Following the above line of thinking, this paper proposes a new double-layer cubature Kalman filter (DLCKF) algorithm which uses the weighted sampling points to represent the posterior density function at the previous moment. Besides, the weighted sampling points and their corresponding weights are updated by the inner CKF and the latest measurements, respectively. In addition, each sampling point is weighted fusion to attain the initial value. Finally, the final value is obtained by employing the outer CKF to update the initial value. The proposal algorithm is applicable for large process noise and low measurement noise.

This paper is an extended and revised version of the conference paper [33]. It provides new simulations, improvements, and further details, including mainly the following: (a) more detailed derivations and discussions about the correlated algorithms are added; (b) the fundamentals of the proposed algorithm is revised to be more comprehensible; (c) the weight of the updated cubature point in the outer CKF of the DLCKF is added as well as the flowsheet of the DLCKF is revised for better formulations; (d) four algorithms (UKF, ICKF, UPF, RUCKF) and applications in real-world scenario are added in the simulation to demonstrate the excellent performance of the DLCKF.

The rest of this paper is organized as follows. In Section 2, the fundamentals of the CKF and CPF are briefly reviewed. Section 3 presents a new DLCKF algorithm based on the CKF. Then, simulation results are provided to demonstrate the performance of the proposed algorithm in Section 4. At last, some conclusions are given in Section 5.

## 2. Problem Formulation

### 2.1. Cubature Rule

The problem of non-linear integrating in Gauss estimation can be expressed as

(1) $$I(f)=\int_{R^{n_{x}}}f\left(x\right)exp\left(-x^{T}x\right)dx$$

In order to compute the above numerical integration, the following two steps are taken. First, the integration is transformed into a form of spherical-radial cubature, and then three-degree spherical-radial cubature rule is designed. The specific method is as follows:

In the cubature rule, the key is the transformation from vector *x* to radius *r* and direction vector *y*. Supposing x=ry, $y^{T}y=1$,then $x^{T}x=r^{2},r\in \left[0,+\infty \right)$. So, Equation (1) in the spherical-radial coordinate system can be expressed as

(2) $$I(f)=\int_{0}^{\infty }\int_{U_{n_{x}}}f\left(ry\right)r^{n-1}exp\left(-r^{2}\right)d\left(\sigma y\right)dr$$

where $U_{n_{x}}$ denotes sphere surface with radius 1, that is $U_{n_{x}}=\left\{y\in R^{n_{x}}|y^{T}y=1\right\}$, σ(·) represents spherical measure. So, Equation (2) can be rewritten. $m_{r}$ points and $m_{s}$ points are generated by the radial rule and the spherical rule respectively [18].

So, $m_{r}\times m_{s}$ points produced by spherical-radial cubature rule can be expressed as

(3) $$I(f)=\int_{R^{n_{x}}}f\left(x\right)exp\left(-x^{T}x\right)dx\approx \sum_{j=1}^{m_{s}}\sum_{i=1}^{m_{r}}a_{i}b_{j}f\left(r_{i}y_{j}\right)$$

For the three-degree spherical-radial cubature rule, there are $m_{r}=1$ and $m_{s}=2n_{x}$. Therefore, 2n points are needed. Accordingly, calculating a standard Gauss weight integral can be expressed as

(4) $$I(f)=\int_{R^{n_{x}}}f\left(x\right)N\left(x;0,I_{n_{x}}\right)dx\approx \sum_{i=1}^{m}w_{i}f\left(\chi_{i}\right)$$

where $m=2n_{x}$;$\chi_{i}=\sqrt{m/2}{\left[1\right]}_{i}$;$w_{i}=1/m,\left(i=1,2,\ldots ,m\right)$, $\left[1\right]\in R^{n_{x}}$ and

(5) $${\left[1\right]}_{i}\in \left\{\left|\begin{array}{c} \begin{array}{c} 1\\ 0 \end{array}\\ \vdots \\ 0 \end{array}\right|,\cdots ,\left|\begin{array}{c} \begin{array}{c} 0\\ 0 \end{array}\\ \vdots \\ 1 \end{array}\right|,\left|\begin{array}{c} \begin{array}{c} -1\\ 0 \end{array}\\ \vdots \\ 0 \end{array}\right|,\cdots ,\left|\begin{array}{c} \begin{array}{c} 0\\ 0 \end{array}\\ \vdots \\ -1 \end{array}\right|\right\}$$

Given $x∼N(x;\hat{x},P)$,$P=SS^{T}$,then the Equation (8) can be transformed into

(6) $$I(f)=\int_{R^{n_{x}}}f\left(x\right)N\left(x;\hat{x},P\right)dx\approx \sum_{i=1}^{m}w_{i}f\left(S\chi_{i}+\hat{x}\right)$$

### 2.2. Cubature Kalman Filter

Consider the discrete-time state stochastic system, whitch is also used in [20,24]:

(7) $$x_{k}=f\left(x_{k-1}\right)+\omega_{k-1}$$

(8) $$z_{k}=h\left(x_{k}\right)+v_{k},$$

where *k* denotes the discrete time index, $x_{k}\in R^{m}$ is the state vector, $z_{k}\in R^{n}$ is the measurement vector. $w_{k-1}\in R^{m}$ and $v_{k}\in R^{n}$ denote the process noise vector and the measurement noise vector. The process noise and the measurement noise are zero-mean random variables with known probability density functions. The functions f(·) and h(·) are assumed to be known.

The process of the CKF Algorithm 1 is summarized as follows:

**Algorithm 1** The CKF algorithm [8] for one round
**Input:**${\hat{x}}_{k-1}$, $P_{k-1}$, *f*, *h*
**Output:**${\hat{x}}_{k}$, $P_{k}$
1:Time update:  Generate *m* cubature points and corresponding weights:  $\xi_{j,k-1}=chol\left(P_{k-1}\right)\chi_{i}+{\hat{x}}_{k-1}$, $w_{j}=1/m$  Propagate each cubature point:  $\xi_{j,k\left|k-1\right.}=f\left(\xi_{j,k-1}\right)$
  Obtain predicted state and predicted covariance:  ${\hat{x}}_{k\left|k-1\right.}=\sum_{j=1}^{m}w_{j}\xi_{j,k\left|k-1\right.}$, $P_{k\left|k-1\right.}=\sum_{j=1}^{m}{w_{j}\left(\xi_{j,k|k-1}-{\hat{x}}_{k|k-1}\right)\left(\xi_{j,k|k-1}-{\hat{x}}_{k|k-1}\right)}^{T}+Q$  2:Measure update:  Generate *m* integration points and corresponding weights:  $\zeta_{j,k\left|k-1\right.}=chol\left(P_{k\left|k-1\right.}\right)\chi_{i}+{\hat{x}}_{k\left|k-1\right.}$, $w_{j}=1/m$  Propagate the integration point:  $\varepsilon_{j,k\left|k-1\right.}=h\left(\zeta_{j,k\left|k-1\right.}\right)$  3:Obtain predicted measurement, covariance and mutual covariance:  ${\hat{z}}_{k\left|k-1\right.}=\sum_{j=1}^{m}w_{j}\varepsilon_{j,k\left|k-1\right.}$, $P_{zz}=\sum_{j=1}^{m}{w_{j}\left(\varepsilon_{j,k|k-1}-{\hat{z}}_{k|k-1}\right)\left(\varepsilon_{j,k|k-1}-{\hat{z}}_{k|k-1}\right)}^{T}+R$, $P_{xz}=\sum_{j=1}^{m}{w_{j,i}\left(\xi_{j,k|k-1}-{\hat{x}}_{k|k-1}\right)\left(\varepsilon_{j,k|k-1}-{\hat{z}}_{k|k-1}\right)}^{T}$  4:Obtain state estimation and corresponding covariance:  ${\hat{x}}_{k}={\hat{x}}_{k|k-1}+K_{k}\left(z_{k}-{\hat{z}}_{k|k-1}\right)$, $P_{k}=P_{k|k-1}-K_{k}P_{zz}{K_{k}}^{T}$


### 2.3. Cubature Particle Filter

Based on particle filter (PF), the cubature particle filter (CPF) [29,30] adopts the CKF as the importance probability distribution function. Although it is suitable for Gaussian systems and non-Gaussian systems, it may result in heavy computational load owing to the use of a large number of particles. The process of the CPF can be summarized as follows:

(1) Based on the prior probability distribution $p\left(x{}_{0}\right)$, select *N* particles ${\left\{x_{0}^{\left(i\right)}\right\}}_{i=1}^{N}$;
(2) Use CKF to update each particle;
(3) Use latest measurements to update each weight $w_{k}^{\left(i\right)}=p\left(y_{k}\left|{\hat{x}}_{k}^{\left(i\right)}\right.\right)p\left({\hat{x}}_{k}^{\left(i\right)}\left|x_{k-1}^{\left(i\right)}\right.\right)/$$\pi \left({\hat{x}}_{k}^{\left(i\right)}\left|x_{k-1}^{\left(i\right)}\right.,y_{k}\right)$, and then normalize them;
(4) Calculate the number of effective particles $N_{eff}$, if $N_{eff}<N_{threshold}$, resample;
(5) Obtain state estimation ${\hat{x}}_{k}$ and covariance $P_{k}$ by accumulating particles at time *k*;
(6) Get state estimation at each time by repeating steps (2) to (5).

## 3. Double-Layer Cubature Kalman Filter (DLCKF)

Since CPF needs a large number of random particles to approximate the posterior density function of the state, it results in heavy computational load. In contrast to CPF, the proposed DLCKF uses deterministic weighted sampling points to approximate the posterior density function. The core idea of DLCKF is using the inner CKF to update each sampling point, and then the weight of each sampling point is updated with the latest measurement. Next, weighted summation of the updated sampling points is performed to obtain the initial state estimation for the next time. The obtained initial state estimation is used as the predictive value to run the outer CKF algorithm. Final, the state estimation is obtained.

The proposed DLCKF consists of inner-layer CKF and outer-layer CKF. The process of DLCKF is given as follows:

Initial value is ${\hat{x}}_{0}=E\left[x_{0}\right]$, and initial covariance can be expressed as $P_{0}=\mathrm{cov}\left(x_{0}\right)$

At time k−1, the $2n_{x}$ cubature points ${\left\{\xi_{i,k-1}\right\}}_{i=1}^{2n_{x}}$ and the corresponding weight value ${\left\{w_{i}\right\}}_{i=1}^{2n_{x}}$ are obtained by cubature rule. Then the inner-layer CKF is used to update each cubature point.

**The inner-layer CKF:**

The cubature point $\chi_{j,i}=\sqrt{m/2}{\left[1\right]}_{j,i}$ with corresponding weight $w_{j,i}=1/m$ are obtained by three-degree spherical-radial cubature rule, where *m* denotes the total number of cubature points, which is twice the state dimension, that is $m=2n_{x}$, and $n_{x}$ is the dimension of the state. $\left[1\right]\in R^{n_{x}}$ can be written as

(9) $${\left[1\right]}_{j,i}\in \left\{\left|\begin{array}{c} \begin{array}{c} 1\\ 0 \end{array}\\ \vdots \\ 0 \end{array}\right|,\cdots ,\left|\begin{array}{c} \begin{array}{c} 0\\ 0 \end{array}\\ \vdots \\ 1 \end{array}\right|,\left|\begin{array}{c} \begin{array}{c} -1\\ 0 \end{array}\\ \vdots \\ 0 \end{array}\right|,\cdots ,\left|\begin{array}{c} \begin{array}{c} 0\\ 0 \end{array}\\ \vdots \\ -1 \end{array}\right|\right\}$$

Then, based on the cubature point ${\left\{\chi_{j,i}\right\}}_{j=1}^{m}$ and its weight ${\left\{w_{j,i}\right\}}_{j=1}^{m}$, the cubature point ${\left\{\xi_{j,i,k-1}\right\}}_{j=1}^{m}$ of the inner-layer CKF is obtained. Then, we have the time update and measurement update steps of the inner-layer CKF as follows.

Time update:

(10) $$S_{i,k-1}=chol\left(P_{i,k-1}\right)$$

(11) $$\xi_{j,i,k-1}=S_{i,k-1}\cdot \chi_{j,i}+\xi_{i,k-1}$$

(12) $$\xi_{j,i,k|k-1}=f\left(\xi_{j,i,k-1}\right)+\omega_{j,i,k-1}$$

(13) $${\hat{x}}_{i,k|k-1}=\sum_{j=1}^{m}w_{j,i}\xi_{j,i,k|k-1}$$

(14) $$P_{i,k|k-1}=\sum_{j=1}^{m}{w_{j,i}\left(\xi_{j,i,k|k-1}-{\hat{x}}_{i,k|k-1}\right)\left(\xi_{j,i,k|k-1}-{\hat{x}}_{i,k|k-1}\right)}^{T}+Q$$

Measurement update:

(15) $$S_{i,k|k-1}=chol\left(P_{i,k|k-1}\right)$$

(16) $$\zeta_{j,i,k|k-1}=S_{i,k|k-1}\cdot \chi_{j,i}+{\hat{x}}_{i,k|k-1}$$

(17) $$\varepsilon_{j,i,k|k-1}=h\left(\zeta_{j,i,k|k-1}\right)+v_{j,i,k|k-1}$$

(18) $${\hat{z}}_{i,k|k-1}=\sum_{j=1}^{m}w_{j,i}\varepsilon_{j,i,k|k-1}$$

(19) $$P_{i,zz}=\sum_{j=1}^{m}{w_{j,i}\left(\varepsilon_{j,i,k|k-1}-{\hat{z}}_{i,k|k-1}\right)\left(\varepsilon_{j,i,k|k-1}-{\hat{z}}_{i,k|k-1}\right)}^{T}+R$$

(20) $$P_{i,xz}=\sum_{j=1}^{m}{w_{j,i}\left(\xi_{j,i,k|k-1}-{\hat{x}}_{i,k|k-1}\right)\left(\varepsilon_{j,i,k|k-1}-{\hat{z}}_{i,k|k-1}\right)}^{T}$$

(21) $$K_{i,k}=P_{i,xz}{P_{i,zz}}^{-1}$$

(22) $${\hat{x}}_{i,k}={\hat{x}}_{i,k|k-1}+K_{i,k}\left(z_{i,k}-{\hat{z}}_{i,k|k-1}\right)$$

(23) $$P_{i,k}=P_{i,k|k-1}-K_{i,k}P_{i,zz}{K_{i,k}}^{T}$$

**The outer-layer CKF:**

After the cubature point updated by the inner CKF, its weight can be caculated as follows.

(24) $$w_{i}=w_{i}\frac{p\left(z_{k}|{\hat{x}}_{i,k}\right)p\left({\hat{x}}_{i,k}|{\hat{x}}_{i,k-1}\right)}{q\left({\hat{x}}_{i,k}|z_{1:k}\right)}$$

Normlize the weight as follows:

(25) $$w_{i}=\frac{w_{i}}{\sum_{i=1}^{2n_{x}}w_{i}}$$

Then, the initial estimation with corresponding covariance at time k can be expressed as

(26) $${\hat{x}}_{k|k-1}=\sum_{i=1}^{2n_{x}}w_{i}{\hat{x}}_{i,k}$$

(27) $$P_{k|k-1}=\sum_{i=1}^{2n_{x}}{w_{i}\left({\hat{x}}_{i,k}-{\hat{x}}_{k|k-1}\right)\left({\hat{x}}_{i,k}-{\hat{x}}_{k|k-1}\right)}^{T}+Q$$

Based on ${\hat{x}}_{k|k-1}$ and $P_{k|k-1}$, the cubature points can be updated as:

(28) $$S_{k|k-1}=chol\left(P_{k|k-1}\right)$$

(29) $$\zeta_{i,k|k-1}=S_{k|k-1}\cdot \chi_{i}+{\hat{x}}_{k|k-1}$$

(30) $$\varepsilon_{i,k|k-1}=h\left(\zeta_{i,k|k-1}\right)+v_{i,k|k-1}$$

(31) $${\hat{z}}_{k|k-1}=\sum_{i=1}^{2n_{x}}w_{i}\varepsilon_{i,k|k-1}$$

(32) $$P_{zz}=\sum_{i=1}^{2n_{x}}{w_{i}\left(\varepsilon_{i,k|k-1}-{\hat{z}}_{k|k-1}\right)\left(\varepsilon_{i,k|k-1}-{\hat{z}}_{k|k-1}\right)}^{T}+R$$

(33) $$P_{xz}=\sum_{i=1}^{2n_{x}}{w_{i}\left({\hat{x}}_{i,k}-{\hat{x}}_{k|k-1}\right)\left(\varepsilon_{i,k|k-1}-{\hat{z}}_{k|k-1}\right)}^{T}$$

(34) $$K_{k}=P_{xz}{P_{zz}}^{-1}$$

(35) $${\hat{x}}_{k}={\hat{x}}_{k|k-1}+K_{k}\left(z_{k}-{\hat{z}}_{k|k-1}\right)$$

(36) $$P_{k}=P_{k|k-1}-K_{k}P_{zz}{K_{k}}^{T}$$

Repeating Equations from Equation (10) to (36) state estimation $\hat{x}$ of the proposed DLCKF at each time can be obtained.

**Figure 1:**
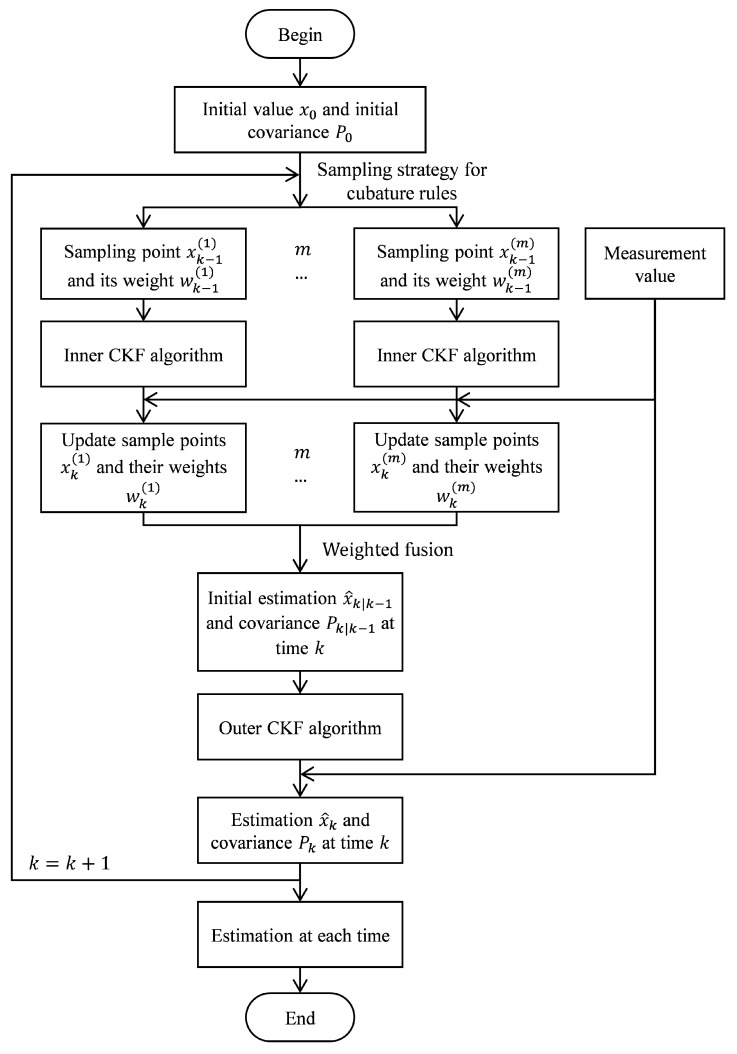
The flowsheet of DLCKF.

The process of the proposed DLCKF algorithm is summarized as Figure 1:

## 4. Simulation Experiments

In this section, to illustrate the effectiveness of the proposed algorithm, the DLCKF is compared with CKF [8,9,10], UKF [6,7], ICKF [22], CPF [29,30], UPF [34,35] and RUCKF [24] in two classical filtering applications and a real-world scenario. The root mean squared error (RMSE) is used to indicate the estimation performance. All simulations are executed on an Intel Core i5-4200H CPU 2.80 GHz personal computer with 8 GB of random-access memory.

### 4.1. Single-Dimensional Scenario

**Figure 2:**
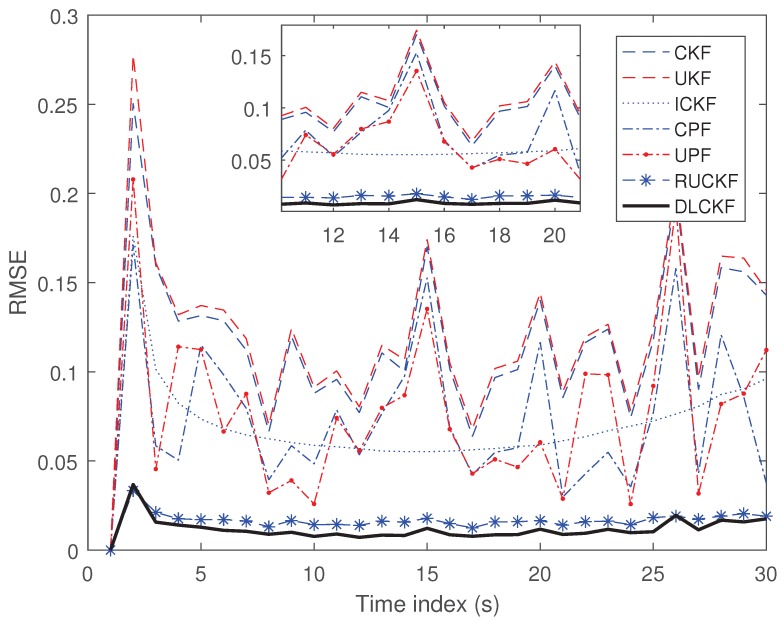
RMSE of 300 Monte Carlo simulations.

We first consider the following univariate nonstationary growth model, which has been also used in [36,37] for its high nonlinearity. Its state space model can be written as:

(37) $$x_{k+1}=0.5x_{k}+\sin\left(0.04\pi k\right)+1+\omega_{k}$$

(38) $$z_{k+1}=0.2x_{k+1}^{2}+v_{k+1}$$

where process noise $\omega_{k}$ obeys gamma distribution $Ga\left(3,2\right)$, and measurement noise $v_{k+1}$ obeys Gaussian distribution with mean of 0 with variance of $R=10^{-5}$. The initial position is $x_{0}=3$. The iterative number of ICKF is 10 and the particle number of the CPF as well as UPF is set as 100. The Monte Carlo times are 300 and the simulation time is 30 s. The simulation results are shown in Figure 2.

It can be seen from Figure 2 that the RMSE of the CKF is similar to that of the UKF because they both have three-order accuracy of Gaussian distribution. The performance of the CPF and the UPF is slightly better than those of the CKF and the UKF, since the former make certain improvements to the latter. The ICKF shows better performance than all above methods to some extent as the result of its iterative process which applies state estimation and covariance of each timestep as the initial value. The RUCKF is adaptable in single-dimensional scenario and therefore its RMSE is low. The proposed algorithm performs best at each timestep, which indicates the improvement of the DLCKF is more significant than that of others.

The number of the particles in the CPF and UPF is gradually increased from 100 to 500. The single run time and average RMSE of each algorithm are shown in Table 1.

Table 1 illustrates the performance of the run time and average RMSE of each algorithm. It can be seen that the CKF and the UKF take the least time cost, and the CPF and the UPF takes the longest time cost. In the CPF and the UPF, as the number of particles increases, the run time gradually becomes longer. In terms of RMSE, the DLCKF is much smaller than that of the other five methods. The ICKF and RUCKF have lower RMSE than the CKF due to their iteration. As the number of the CPF and UPF increases, their RMSE also decreases. When the particle number is 500, the RMSE of the CPF and the UPF is still twice that of the DLCKF. The above results all indicate that in single-dimensional scenario, the proposed DLCKF has better performance than the other algorithms.

### 4.2. Multi-Dimensional Scenario

Consider tracking a 2-D uniform linear motion [38], while the state space model expressed as:

(39) $$x_{k+1}=Fx_{k}+\omega_{k}$$

(40) $$z_{k+1}=h\left(x_{k+1}\right)+v_{k+1}$$

where, $x_{k}=\left[x_{k},{\dot{x}}_{k},y_{k},{\dot{y}}_{k}\right]$ is the state variable that represents the position and velocity of the *x* axis and *y* axis, respectively, the process noise $\omega_{k}$ obeys a Gaussian distribution with zeros mean and variance matrix *Q*, and *F* and *Q* are written as

(41) $$F=\left[\begin{array}{cccc} 1 & T & 0 & 0\\ 0 & 1 & 0 & 0\\ 0 & 0 & 1 & T\\ 0 & 0 & 0 & 1 \end{array}\right]$$

(42) $$Q=q\left[\begin{array}{cccc} T^{3}/3 & T^{2}/2 & 0 & 0\\ T^{2}/2 & T & 0 & 0\\ 0 & 0 & T^{3}/3 & T^{2}/2\\ 0 & 0 & T^{2}/2 & T \end{array}\right]$$

In Equation (40), $z_{k+1}={\left[r_{k+1},\theta_{k+1}\right]}^{T}$ denotes the measurement variable, which respectively represents the radial distance and azimuth angle of the target, $v_{k+1}$ denotes the measurement noise, which is flicker noise and can be expressed as:

(43) $$\begin{array}{c} p\left(v_{k+1}\right)=\left(1-\varepsilon \right)p_{1}\left(v_{k+1}\right)+\varepsilon p_{2}\left(v_{k+1}\right)\\ =\left(1-\varepsilon \right)N\left(v_{k+1};0,R_{1}\right)+\varepsilon N\left(v_{k+1};0,R_{2}\right) \end{array}$$

The measurement equation h(·) can be written as:

(44) $$h\left(x_{k+1}\right)={\left[\begin{array}{cc} \sqrt{x_{k}^{2}+y_{k}^{2}} & \arctan(y_{k},x_{k}) \end{array}\right]}^{T}$$

In Equation (43), $R_{1}$ and $R_{2}$ can be expressed as

(45) $$R_{1}=\left[\begin{array}{cc} \sigma_{1r}^{2} & 0\\ 0 & \sigma_{1\varepsilon }^{2} \end{array}\right]$$

(46) $$R_{2}=\left[\begin{array}{cc} \sigma_{2r}^{2} & 0\\ 0 & \sigma_{2\varepsilon }^{2} \end{array}\right]$$

In this simulation, the initial value of the state is (20,000 m, −160 m/s, 40,000 m, −150 m/s). The iterative times of the ICKF is set as 10, the particle number of the CPF and the UPF is 300. Other parameters are set as Table 2. The simulation time is 100 s, and the Monte Carlo times is set as 300.

The RMSE of position can be expressed as:

(47) $$\mathrm{RMSE}=\sqrt{{\mathrm{RMSE}}_{x}^{2}+{\mathrm{RMSE}}_{y}^{2}}$$

**Figure 3:**
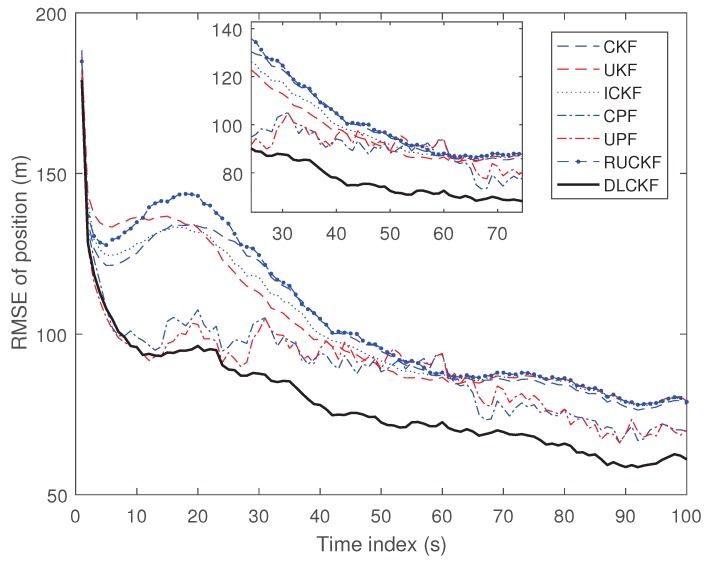
RMSE of position.

The result of simulation is as Figure 3.

Figure 3 shows the position RMSE of each algorithm. It can be seen that the ICKF, the CPF and the UPF are superior to the CKF and the UKF and the RUCKF is not suitable for the multi-dimensional scenario. The performance of the DLCKF is the best. As a result, the RMSE of the DLCKF is the lowest in the above filtering methods. This also shows that the proposed DLCKF performs greatly on the 2-D uniform linear motion.

In the 2-D uniform linear motion, the number of the particles in the CPF and UPF increases gradually from 300 to 1000. The single run time and the average RMSE of each algorithm are shown as Table 3.

From Table 3, it can be seen that in terms of the running time, the proposed DLCKF is slightly longer than that of the CKF and UKF, but it is far less than that of the CPF and UPF. In the CPF and UPF, as the number of particles increases, their computational time also increases. In terms of average RMSE, as the particle number of the CPF and UPF increases, their average RMSE also gradually reduces. However, compared with the DLCKF, the RMSE of the CPF and UPF is still sizable. All of this indicate that the proposed DLCKF has a great estimation effect in 2-D uniform linear motion.

### 4.3. Real-World Scenario

**Figure 4:**
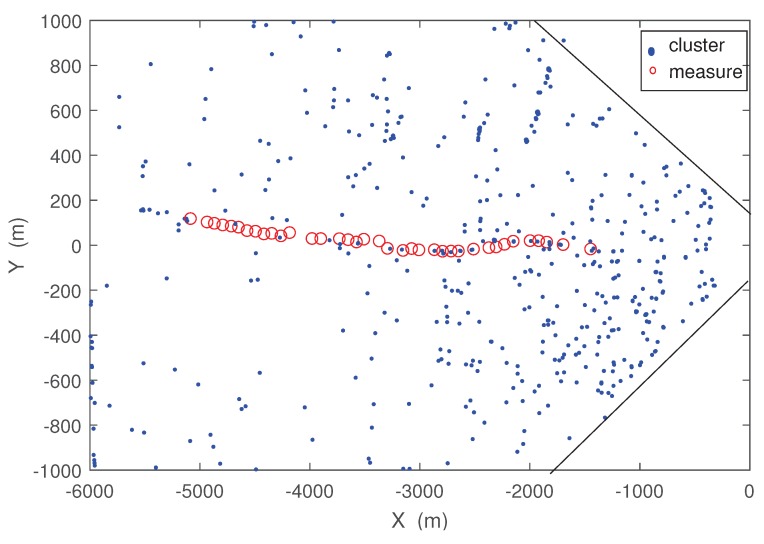
Real-world scenario.

Measurement data of the real-world scenario is obtained by a real radar sensor. A given target appears in a 6 km × 2 km area covered by the sensor. The given target is a car driving along a road in this area with a constant velocity. The initial position of the target is (−1443.6 m, −18.5454 m ), the running speed of the target is almost 32 km/h, and scanning period is 8 s. All illustration of the sensor and the target is presented in Figure 4.

In this experiment, a 2-D uniform linear motion model is applied and measurement value comes from the real radar sensor. We manually wipe off the measurements which are not from the certain target we concerned.

**Figure 5:**
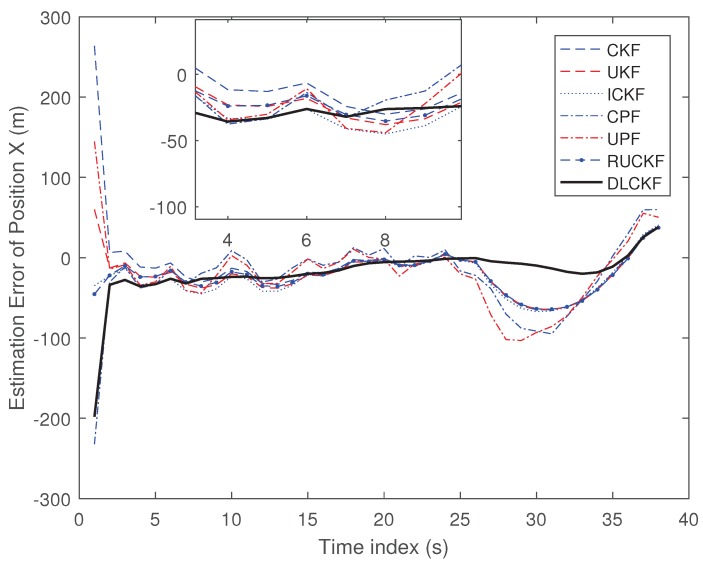
Estimation Error of Position X (m).

**Figure 6:**
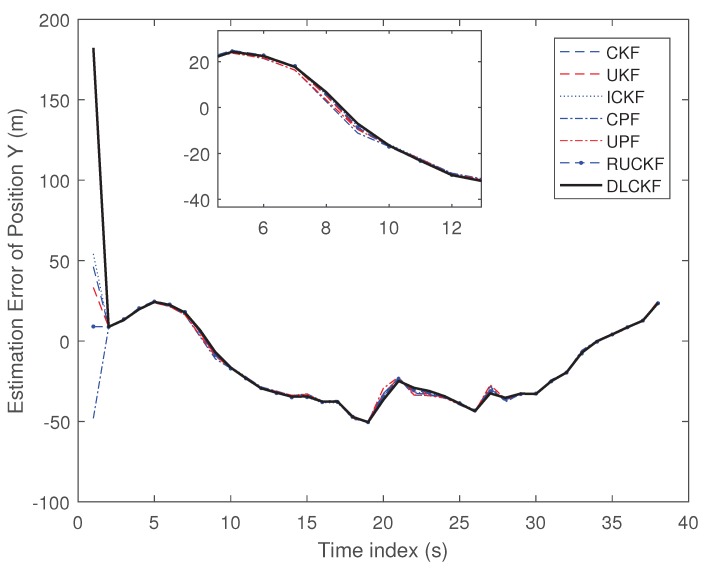
Estimation Error of Position Y (m).

The results of experiments are shown in Figure 5 and Figure 6.

Figure 5 and Figure 6 shows the estimation error of position X, Y of each algorithm in real-world scenario. It can be seen that the CPF and the UPF are superior to the CKF, the UKF, the ICKF and the RUCKF. The DLCKF performs best, which indicates that the proposed DLCKF can obtain great estimation in the real-world scenario.

In this simulation, the number of the particles in the CPF and UPF is 300. The single run time and the average estimation error (AEE) of position X, Y of each algorithm are shown as Table 4.

From Table 4, it can be seen that in terms of the running time, the proposed DLCKF has slightly longer timecost than that of the CKF and UKF, but it is far less than that of the CPF and UPF. In terms of average estimation error of position X and Y, the CPF and the UPF perform better compared with the CKF and the UKF, and the DLCKF performs best. All of those demonstrate that the proposed DLCKF has better performance than other algorithms in real-world scenario.

## 5. Conclusions

The DLCKF that is based on CKF is proposed in this paper, which uses the cubature points to approximate the prior density function of the state. The cubature points are updated by the inner-layer CKF, meanwhile, the weight of each cubature point in the outer-layer CKF is updated with the latest measurement. Finally, the state estimations at each time are obtained through the update mechanism of the outer-layer CKF. The experiments results show that in one-dimensional and multi-dimensional simulation scenarios, the DLCKF performs best compared with the CKF, UKF, ICKF, CPF, UPF and RUCKF. In addition, the superiority of the DLCKF is its effectiveness of in a more challenging environment, which brings great challenges to classic algorithms. The experiments in real-world scenario confirm it. All of those indicate that the proposed DLCKF proposed can obtain an outstanding estimation accuracy with low time cost, compared to classic algorithms.

## Figures and Tables

**Table 1:** The run time and the average RMSE of each algorithm.

Algorithm	Run Time (s)	Average RMSE (m)
CKF	0.0013	0.0335
UKF	0.0010	0.0344
ICKF	0.0026	0.0186
CPF(100)	0.5057	0.0241
CPF(200)	1.0398	0.0172
CPF(300)	1.6181	0.0113
CPF(400)	2.2107	0.0111
CPF(500)	2.8181	0.0106
UPF(100)	0.5035	0.0257
UPF(200)	1.0319	0.0146
UPF(300)	1.6009	0.0139
UPF(400)	2.2084	0.0122
UPF(500)	2.8250	0.0103
RUCKF	0.0025	0.0044
DLCKF	0.0035	0.0039

**Table 2:** The Simulation parameters.

Parameter	*T*	*q*	$\sigma_{1r}$	$\sigma_{1\varepsilon }$	$\sigma_{2r}$	$\sigma_{2\varepsilon }$	ε
Value	1 s	1 m${}^{2}$/s${}^{3}$	20 m	0.2${}^{\circ }$	200 m	2${}^{\circ }$	0.1

**Table 3:** Performance of each algorithm.

Algorithm	Run Time (s)	Average RMSE (m)
CKF	0.0187	102.8858
UKF	0.0190	97.3787
ICKF	0.0326	101.1720
CPF (300)	9.8903	89.3956
CPF (400)	12.7624	88.4210
CPF (500)	16.0016	87.0589
CPF (600)	19.0964	86.0255
CPF (700)	22.3376	84.6944
CPF (800)	25.6078	83.8060
CPF (900)	28.7884	83.1365
CPF (1000)	31.9711	83.0590
UPF (300)	9.9026	89.2771
UPF (400)	12.8477	87.3480
UPF (500)	16.0309	86.9156
UPF (600)	19.1404	85.6700
UPF (700)	22.3845	83.8380
UPF (800)	25.6139	83.4147
UPF (900)	28.8655	83.0882
UPF (1000)	32.3971	82.8012
RUCKF	0.1140	104.9231
DLCKF	0.2189	79.7820

**Table 4:** Performance of each algorithm.

Algorithm	Run Time (s)	AEE of Position X (m)	AEE of Position Y (m)
CKF	0.0099	−20.7860	−14.8852
UKF	0.0150	−21.0891	−15.3066
ICKF	0.0710	−22.6846	−14.8039
CPF	7.8441	−19.6904	−12.5453
UPF	8.0290	−19.9458	−13.3300
RUCKF	0.1504	−21.3505	−15.9280
DLCKF	0.1976	−18.3091	−11.3136

## References

[1] Song Y., Nuske S., Scherer S. (2017). A Multi-Sensor Fusion MAV State Estimation from Long-Range Stereo, IMU, GPS and Barometric Sensors. Sensors.

[2] Mu X., Chen J., Zhou Z., Leng Z., Fan L. (2018). Accurate Initial State Estimation in a Monocular Visual-Inertial SLAM System. Sensors.

[3] Yu Q., Xiong R., Lin C., Shen W., Deng J. (2017). Lithium-Ion Battery Parameters and State-of-Charge Joint Estimation Based on H-Infinity and Unscented Kalman Filters. IEEE Trans. Veh. Technol..

[4] Chen T., Yse F., Ling K.V., Chen X. (2017). Distributed State Estimation Using a Modified Partitioned Moving Horizon Strategy for Power Systems. Sensors.

[5] Reif K., Gunther S., Yaz E., Unbehauen R. (2000). Stochastic stability of the continuous-time extended Kalman filter. IEE Proc. Control Theory Appl..

[6] Julier S.J., Uhlmann J.K. (2004). Unscented Filtering and Nonlinear Estimation. Proc. IEEE.

[7] Peng S., Chen C., Shi H., Yao Z. (2017). State of Charge Estimation of Battery Energy Storage Systems Based on Adaptive Unscented Kalman Filter with a Noise Statistics Estimator. IEEE Access.

[8] Arasaratnam I., Haykin S. (2009). Cubature Kalman Filters. IEEE Trans. Autom. Control.

[9] Liu H., Wu W. (2017). Strong Tracking Spherical Simplex-Radial Cubature Kalman Filter for Maneuvering Target Tracking. Sensors.

[10] He R., Chen S., Wu H., Hong L., Chen K. (2018). Stochastic Feedback Based Continuous-Discrete Cubature Kalman Filtering for Bearings-Only Tracking. Sensors.

[11] Arulampalam M.S., Maskell S., Gordon N., Arulampalam M.S., Maskell S., Gordon N., Clapp T. (2002). A tutorial on particle filters for online nonlinear/non-Gaussian Bayesian tracking. IEEE Trans. Signal Process..

[12] Genshiro K. (2014). Computational aspects of sequential Monte Carlo filter and smoother. Ann. Inst. Stat. Math..

[13] Li X.R., Jilkov V.P. A survey of maneuvering target tracking: approximation techniques for nonlinear filtering. Proceedings of the 2004 SPIE Conference on Signal and Data Processing of Small Targets.

[14] Li X.R., Jilkov V.P. (2010). A survey of maneuvering target tracking-part VIb: Approximate nonlinear density filtering in mixed time. Proc. SPIE Int. Soc. Opt. Eng..

[15] Kozlov V.V., Furta S.D. (1996). Lyapunov’s first method for strongly non-linear systems. J. Appl. Math. Mech..

[16] Simon D.J. (2006). Optimal State Estimation: Kalman, H Infinity, and Nonlinear Approaches.

[17] Li T.C., Su J.Y., Liu W., Corchado J.M. (2017). Approximate Gaussian conjugacy: Parametric recursive filtering under nonlinearity, multimodality, uncertainty, and constraint, and beyond. Front. Inf. Technol. Electron. Eng..

[18] Hao Y.L., Yang J.W., Chen L., Hao J.H. (2012). Square root cubature Kalman filter. J. Proj. Rocket. Missiles Guid..

[19] Jia B., Xin M., Cheng Y. (2013). High-Degree Cubature Kalman Filter.

[20] Zhang Y., Huang Y., Li N., Zhao L. (2015). Embedded cubature Kalman filter with adaptive setting of free parameter. Signal Process..

[21] Zhang Y., Li N., Zhao L., Huang Y. (2015). Interpolatory cubature Kalman filters. IET Control Theory Appl..

[22] Mu J., Cai Y. (2011). Iterated cubature Kalman filter and its application. Syst. Eng. Electron..

[23] Zanetti R. (2012). Recursive Update Filtering for Nonlinear Estimation. IEEE Trans. Autom. Control.

[24] Huang Y., Zhang Y., Li N., Zhao L. (2015). Design of Sigma-Point Kalman Filter with Recursive Updated Measurement. Circuits Syst. Signal Process..

[25] Li T.C., Bolic M., Djuric P.M. (2015). Resampling Methods for Particle Filtering: Classification, implementation, and strategies. IEEE Signal Process. Mag..

[26] Maurelli F., Szymon K., Petillot Y., Salvi J. A Particle Filter Approach for AUV Localization. Proceedings of the Oceans 2008.

[27] Bravo F.G., Vale A., Ribeiro M.I. (2007). Navigation strategies for cooperative localization based on a particle-filter approach. Integr. Comput. Aided Eng..

[28] Wei W., Gao S., Zhong Y., Gu C., Hu G. (2018). Adaptive Square-Root Unscented Particle Filtering Algorithm for Dynamic Navigation. Sensors.

[29] Sun F., Tang L.J. (2011). Cubature particle filter. Syst. Eng. Electron..

[30] Wang D., Yang F., Tsui K.L., Zhou Q., Bae S.J. (2016). Remaining Useful Life Prediction of Lithium-Ion Batteries Based on Spherical Cubature Particle Filter. IEEE Trans. Instrum. Meas..

[31] Ahmad S.U., Antoniou A. Cascade-form multiplierless FIR filter design using orthogonal genetic algorithm. Proceedings of the 2006 IEEE International Symposium on Signal Processing and Information Technology.

[32] Yu C., Lan H., Gu F., Yu F., El-Sheimy N. (2017). A Map/INS/Wi-Fi Integrated System for Indoor Location-Based Service Applications. Sensors.

[33] Yang F., Luo Y., Zheng L., Chen S., Zou J. Double-layer Cubature Kalman Filter. Proceedings of the 2018 International Conference On Control Automation & Information Sciences (ICCAIS 2018).

[34] Merwe R.V.D., Doucet A., Freitas N.D., Wan E.A. (2000). The Unscented Particle Filter. Proceedings of the 13th International Conference on Neural Information Processing Systems.

[35] Wang X., Li T., Sun S. (2017). A Survey of Recent Advances in Particle Filters and Remaining Challenges for Multitarget Tracking. Sensors.

[36] Pruher J., Tronarp F., Karvonen T., Särkkä S., Straka O. Student-t process quadratures for filtering of non-linear systems with heavy-tailed noise. Proceedings of the 2017 20th International Conference on Information Fusion.

[37] Zuo J.Y., Jia Y.N., Zhang Y.Z., Lian W. (2013). Adaptive iterated particle filter. Electron. Lett..

[38] Zheng B., Fu P., Li B., Yuan X. (2018). A Robust Adaptive Unscented Kalman Filter for Nonlinear Estimation with Uncertain Noise Covariance. Sensors.

